# Percutaneous Nephrolithotomy in Rare Bleeding Disorders: A Case Report and Review of the Literature

**DOI:** 10.1089/cren.2016.0105

**Published:** 2016-11-01

**Authors:** Ali Ersin Zumrutbas, Cihan Toktas, Aykut Baser, Omer Levent Tuncay

**Affiliations:** Department of Urology, Pamukkale University School of Medicine, Denizli, Turkey.

**Keywords:** renal stone, percutaneous nephrolithotomy, bleeding disorders, hemophilia

## Abstract

Surgery in patients with congenital or acquired coagulation defects has always been challenging and requires special care with a multidisciplinary approach. Percutaneous nephrolithotomy (PCNL) is a standard procedure performed in patients with kidney stones. Although prone to bleeding more than most of the widely performed surgical procedures, there are not much data regarding PCNL in patients with bleeding disorders or coagulation defects. There are only case reports or series with a small number of patients for the patients with common coagulation defects, including hemophilias. Moreover, there are no reports about PCNL in rare bleeding disorders. In this study, we reported a case referred for kidney stone treatment and diagnosed as Factor VII deficiency during preoperative evaluation. Because it is one of the rare bleeding disorders, we also reviewed the literature in this field.


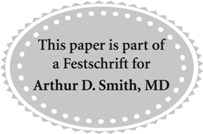


## Introduction

In general, coagulopathies may result from inadequate synthesis of coagulation factor proteins or from inhibition of activated clotting factors by acquired antibodies or medications. Coagulation deficiencies are typically characterized by bleeding after minor or major trauma and surgery and may result in serious morbidity or mortality rate.^1^ Mild or moderate coagulopathies, however, may remain silent until detected coincidentally on routine laboratory screening tests preceding any intervention or surgery in healthcare centers. Hemophilias (A and B) and von Willebrand disease (vWD) constitute the majority of the coagulation disorders due to coagulation factor deficiencies with a prevalence of 1/5000 (Hemophilia A), 1/30,000 (Hemophilia B), and 1% (vWD). The deficiencies of the remaining factors are rarely seen (Table 1).

**Table 1.** Rare Defects of Coagulation and Replacement Therapies Available

*Coagulation factor deficiency*	*Inheritance*	*Prevalence*	*Minimum hemostatic level*	*Replacement sources*
Factor I (Fibrinogen)				
Afibrinogenemia	AR	Rare (<300 families)	50–100 mg/dL	CP, FFP, Fibrinogen concentrate
Dysfibrinogenemia	AD or AR	Rare (>300 variants)		
Factor II (Prothrombin)	AD or AR	Rare (25 kindreds)	30% of normal	FFP, Factor IX complex
Factor V (Labile factor)	AR	1 in 1 million births	25% of normal	FFP
Factor VII	AR	1 in 500,000 births	25% of normal	Recombinant factor VIIa (15–30 mcg/kg), FFP, Factor IX complex
Factor X (Stuart–Prower factor)	AR	1 in 500,000 births	10%–25% of normal	FFP or Factor IX complex
Factor XII (Hageman Factor, prekallikrein, HMWK)	AR	Not available	No treatment necessary	
Factor XIII (Fibrin stabilizing factor)	AR	1 in 3 million births	5% of normal	FFP, CP, or viral attenuated Factor XIII concentrate

Adapted from Schafer.^11^

AD = autosomal dominant; AR = autosomal recessive; CP = cryoprecipitate; FFP = fresh-frozen plasma; HMWK = high-molecular-weight kininogen.

With the recent progress in technology, management of kidney stones is moving toward more minimally invasive options. Extracorporeal shockwave lithotripsy (SWL), percutaneous nephrolithotomy (PCNL), and retrograde intrarenal surgery (RIRS) are the most widely used therapeutic options with wide variety of equipments available.^2^ Determination of the proper treatment differs according to the size and location of the stone, renal anatomy, stone type and hardness, body status and medical condition of the patient, availability of equipment, and choice and experience of the surgeon. Although considered as an “endoscopic” or “minimally invasive” treatment method, PCNL may cause serious complications, including major bleeding, which may require blood transfusion (0%–20%, mean 7%) or rarely may be life threatening.^3,4^ Although it is a kind of surgery performed through a “hole,” passing through the kidney parenchyma, which receives roughly 25% of cardiac output (1.1 L/min), makes it prone to bleeding and more invasive than most of the routine surgical procedures.

Although prone to bleeding more than most of the widely performed surgical procedures, there are not much data regarding PCNL in patients with bleeding disorders or coagulation defects. There are case reports or series with a small number of patients for the patients with hemophilia and vWD and treated for kidney stones.^5–9^ These are mostly about SWL and to our knowledge, no series was published regarding PCNL or RIRS. Moreover, there are no reports about PCNL in rare bleeding disorders.

In this study, we reported the management and course of a 9-year-old girl referred to our clinic for kidney stone treatment and diagnosed as Factor VII deficiency during preoperative evaluation. Because it is one of the rare bleeding disorders, we also reviewed the literature in this field.

## Case Report

**FIG. 1.**
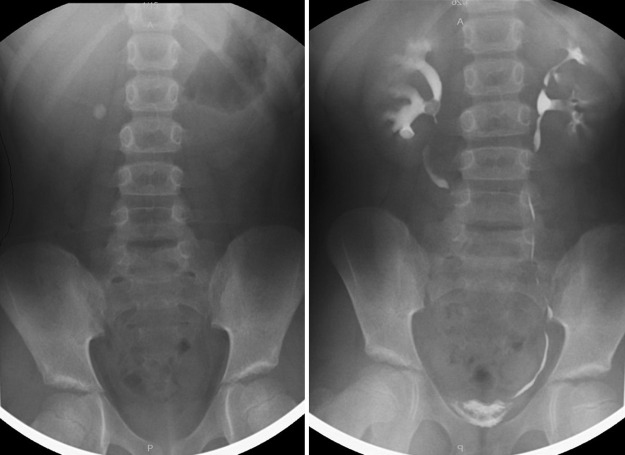
KUB and intravenous urography films showing a stone with a 14 mm diameter in right renal pelvis.

A 9-year-old girl was referred to our department from pediatrics with a diagnosis of right kidney stone. She had right lumbar pain for 1 month when she was admitted to our hospital. She did not have a history of kidney stones or any urinary disease. During the initial evaluation in pediatrics, ultrasonography revealed a stone with a diameter of 14 mm in right renal pelvis and mild hydronephrosis. She had microscopic hematuria and leukocyturia, and her urine culture was negative. Intravenous urography confirmed the findings in ultrasonography with dilated calices and infundibulum (Fig. 1). With the diagnosis of right kidney stone, she was referred to our department for further management. Although all routine biochemical tests were performed previously, we demanded prothrombin time (PT) and activated partial thromboplastin time (aPTT) additionally since these would be necessary for further urologic procedures.

The results for PT and aPTT were 17.3 seconds (normal range: 9.4–13.2 seconds) and 31.7 seconds (normal range: 20–38 seconds), respectively. In the course of pediatric hematology consultation, Factor VII level was found to be 26.5%, which was close to the minimal hemostatic level (25%) (Table 1), and patient was diagnosed as Factor VII deficiency. She had no thrombocytopenia. No abnormalities were found in complete blood count and biochemical laboratory tests. She did not have any history of abnormal bleeding in the past and any family history for coagulation disorders.

As therapeutic options, SWL, RIRS, and mini-PCNL were explained to her family in detail, including therapeutic steps, success rates, and complications. Her family demanded mini-PCNL because of their concerns about the process of stone passage after SWL and the need for more than one procedure (prestenting and stent removal) in RIRS and they wanted the stone removed with minimal intervention. Then the patient was hospitalized and Recombinant Factor VIIa (NovoSeven^®^; Novo Nordisk, Bagsvaerd, Denmark) was started in the early morning, 2 hours before surgery, and repeated every 6 hours for the duration of surgery and postoperative period for 24 hours since Factor VII has a relatively short half-life. The recommended dose for Factor VII replacement is 15 to 30 mcg/kg. Because the weight of the patient was 26 kg, the amount of Factor VII delivered in each application was determined as 0.5 mg.

**FIG. 2.**
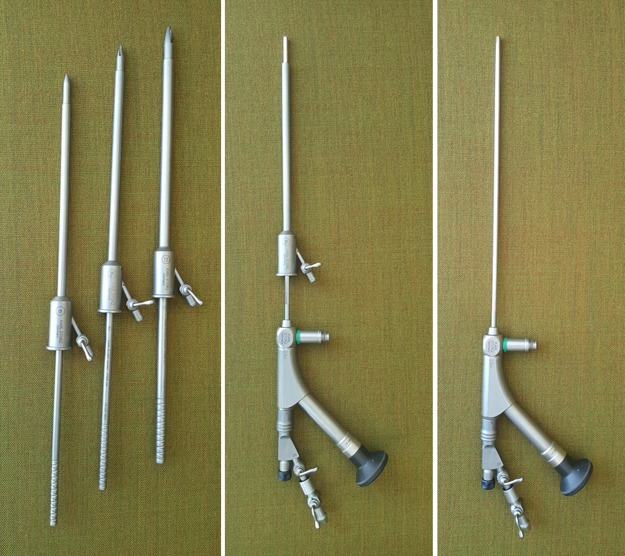
Surgical equipment used in mini-percutaneous nephrolithotomy.

A standard mini-PCNL procedure was performed on the patient. The access site was the lower pole. For tract dilatation, operation, and lithotripsy, a 16.5F single step dilatator (Karl Storz, Germany), a mini nephroscope with a distal diameter of 12F (Karl Storz) (Fig. 2), and a combined pneumatic and ultrasonographic lithotriptor (Swiss LithoClast^®^; EMS, Switzerland) were used. The course of the operation was uneventful. Operation time from the needle access to the end was 40 minutes. The stone was broken with pneumatic lithotriptor, and all the pieces were effectively removed. No residual fragments were observed, which was also confirmed with fluoroscopy. A 10F nephrostomy tube was inserted, which was removed on the morning of the postoperative first day. No major bleeding was observed during the operation and in the postoperative period. The patient was discharged on the postoperative second day. After the cessation of Factor VIIa 24 hours after the initiation of replacement, no hematuria was observed.

## Discussion

In this case report and review, we reported the management and clinical course of a patient in pediatric age group who had a kidney stone and Factor VII deficiency and effectively managed with PCNL. Although there are not enough data in the literature regarding the management of patients with coagulation abnormalities and kidney stones, we tried to review extensively all the studies in this field. We realized that, there are still controversies starting from the preoperative evaluation to the best treatment option.

### Is preoperative testing mandatory for coagulation disorders?

Generally, presurgical testing includes complete blood count, basic metabolic panel, PT, aPTT, electrocardiogram, and chest radiograph. The routine use of a PT/aPTT in a patient not using warfarin or in a patient who gives no prior history of increased bleeding with other surgical procedures is controversial.^10^

A detailed history is important in evaluating a patient for a possible systemic bleeding disorder. In addition to asking the patient about spontaneous bleeding episodes in the past, responses to any hemostatic challenge should be investigated. A bleeding disorder should be suspected if a patient previously experienced high amount of hemorrhage after minor or major surgical procedures such as circumcision, tonsillectomy, labor and delivery, menses, dental procedures, menses and injections, and trauma.^11^ In daily life, excessive bruising or excessive bleeding (i.e., for longer than 3 minutes after brushing teeth, frequent nose bleeds, and prolonged bleeding after simple cuts) may also be a sign of bleeding disorder.^12^ In case of a history of excessive bleeding after a major surgical procedure, any vascular abnormality should be kept in mind for the differential diagnosis. Clinical conditions that predispose patients to bleeding, such as liver and renal dysfunction, should also be noted.^13^

Routine screening of all preoperative patients with all screening tests is not only uninformative but also may be counterproductive if the tests and effort used for the follow-up of these patients cause unnecessary expense and delays in surgery.^1^ In asymptomatic or nonselected patients, coagulation test abnormalities were reported in 0.06% to 21.2% of the patients and led to cancellations or changes in management in 0.0% to 4.0% of the cases with abnormal findings. For selected or indicated patients, abnormal coagulation findings were reported in 3.4% to 29.1% of the patients.^14^

These tests should be useful in patients with a suspicious history for bleeding diathesis and also can be used for screening in patients who are not cooperative, in patients with low socioeconomic status and literacy problems. Screening can also be mandatory in patients who will be undergoing procedures in which even minimal postoperative bleeding could be hazardous.^11^ In our daily practice, we routinely screen all patients in the pediatric age group.

### What should be the next step in case of an abnormal coagulation test?

Basically, there are four simple screening tests used in the initial evaluation of the patients with a suspected bleeding diathesis; bleeding time, platelet count, PT, and aPTT and normal results for all these essentially exclude any clinically significant coagulopathy, with a few exceptions. Bleeding time is usually used to determine any disorders of platelet–vessel wall interactions. It is prolonged in thrombocytopenia, qualitative platelet abnormalities, and platelet–vessel wall interaction defects like vWD and primary vascular disorders. It is not prolonged in factor deficiencies. Because there are problems related to its reliability, some experts recommend replacing this test with specific tests for vWD in the evaluation of patients with suspected coagulation disorders.^11^

The PT measures the integrity of the extrinsic and common pathways of coagulation (Factors VII, X, and V, prothrombin, and fibrinogen), and aPTT measures the integrity of the intrinsic and common pathways (high-molecular-weight kininogen, prekallikrein, factors XII, XI, IX, VIII, X, and V, prothrombin, and fibrinogen).^11^ The PT and aPTT detect only more severe factor deficiencies, usually at levels less than 30% of normal; specific factor levels should be determined if a mild deficiency is suspected. The finding of a prolonged PT and/or aPTT indicates either a deficiency of one or more coagulation factors or the presence of an antibody against the components of the coagulation system. In our patient, a prolonged PT was observed in preoperative screening. Although its level was close to the minimum hemostatic level, pediatric hematology department recommended Factor VII replacement during the operative and early postoperative course of the patient. Treatment of patients with rare bleeding disorders during surgery is a challenge because of the absence of experience, paucity of data, and nonavailability of factor concentrates in some regions.^15^ Table 1 shows the minimum hemostatic level and replacement sources in patients with rare defects of coagulation.

### What should be the ideal approach in kidney stone treatment in patients with rare coagulation disorders?

In our patient, SWL could be a treatment option if her family had accepted this method. Czaplicki and colleagues reported the outcomes of 11 patients with hemophilia who were treated by SWL for urolithiasis.^6^ With the substitution of deficient coagulation factors on the day of treatment and after a total of 25 (1–6/patient) SWL sessions, nine patients (81.8%) discharged stones without any hemorrhagic complications. In a systematic review, including 25 patients, major bleeding complications were observed in 4 patients and SWL was found to be a safe method in the treatment of urinary calculi in the patients with hemophilia.^9^

Another treatment option could be RIRS, which has been extensively used for kidney stones with a tendency to treat larger stones with the invention of new instruments and the high interest and increasing experience of the urologists for this technique. RIRS has also been used for the treatment of pediatric urolithiasis. A recent systematic review, including 6 studies, with a total number of 282 patients (mean age, 7.3 years) reported a stone-free rate of 85.5% after initial ureteroscopy with a postoperative stent inserted in 81.8% of the patients. There were a total of 35 (12.4%) complications with postoperative hematuria observed in 6 patients.^16^ Although most stones in any location can be dealt safely with RIRS, there is a potential need for preoperative and postoperative stenting in children, both of which require a general anesthetic. This was the major concern of the family of the patient in our case, which directed the route of the treatment to PCNL. High success rates with low complication rates were demonstrated with RIRS in pediatric population in recent studies.^17,18^ Although there are no published studies regarding RIRS in patients with rare bleeding disorders, Christman and colleagues reported 7 ureteroscopic and 10 cystoscopic procedures performed effectively in 5 patients having vWD during a 7-year period.^19^ They achieved a stone clearance rate of 100%.

Mokhless and colleagues compared the outcomes of RIRS monotherapy and SWL for stones 10 to 20 mm in preschool children in a prospective randomized study.^20^ They showed that the stone-free rate after a single session treatment was 70% in SWL group and 86.6% in RIRS group. No major complication occurred and no child in either group received blood transfusion. At 3 months, the overall stone-free rate was 93.3% in SWL group and 96.6% in RIRS group.

The last minimally invasive treatment option might be PCNL for the patients with kidney stones and coagulation defects. Unlike RIRS, PCNL has been extensively used in pediatric population with published series, including relatively higher number of patients. When available, dilatators and nephroscopes with the smallest diameter should be used. PCNL performed using small size endoscopes through smaller tracts in diameters ranging from 11F to 20F is named as mini-PCNL or MicroPerc.^21^ Recently, Micro-PCNL or MicroPerc has been described as another minimally invasive PCNL technique performed through a 4.8F all-seeing needle.^22^ PCNL is a challenging procedure in children because of the smaller size of the kidney and the low tolerance to blood loss, which is an important fact in patients with bleeding disorders. Another factor making PCNL challenging in patients with rare bleeding disorders is the fibrinolytic activity displayed by the urinary tract.^15^ In a recent review, initial stone-free rates of mini-PCNL in children were reported between 76% and 94%.^21^ In children, operative parameters associated with increased bleeding were shown as the caliber and number of tracts, duration of surgery, stone burden, and sheath size.^23,24^ A recent multi-institutional study by the Turkish pediatric urology society, which included 1205 renal units in 1157 children treated with PCNL at 16 centers, showed that the rate of bleeding necessitating transfusion was 8%.^25^ In our case, mini-PCNL was performed with the smallest caliber instruments available from a single lower pole access in a short operation time, which helped us to end the operative and postoperative course without any significant bleeding.

## Conclusions

Although there are not enough data and evidence to make a clear conclusion, we suggest that PCNL can be performed safely in patients with rare bleeding disorders. Whenever possible, less invasive options like RIRS should be considered as the first option to decrease the risk of bleeding. Surgical care of the patients with congenital and rare bleeding disorders should only be given by hospitals where cooperation of a multidisciplinary team, including the surgeon and an experienced hematologist, exists. In that setting, adequate sources of replacement and monitoring should always be available.

## Abbreviations Used

AD: autosomal dominant
aPTT: activated partial thromboplastin time
AR: autosomal recessive
CP: cryoprecipitate
FFP: fresh-frozen plasma
HMWK: high-molecular-weight kininogen
KUB: kidney, ureter, bladder
PCNL: percutaneous nephrolithotomy
PT: prothrombin time
RIRS: retrograde intrarenal surgery
SWL: extracorporeal shockwave lithotripsy
vWD: von Willebrand disease
